# Binding of Hemagglutinin and Influenza Virus to a Peptide-Conjugated Lipid Membrane

**DOI:** 10.3389/fmicb.2016.00468

**Published:** 2016-04-07

**Authors:** Teruhiko Matsubara, Rabi Shibata, Toshinori Sato

**Affiliations:** Department of Biosciences and Informatics, Faculty of Science and Technology, Keio UniversityYokohama, Japan

**Keywords:** influenza, hemagglutinin, sialic acid-mimic peptide, lipid bilayer, atomic force microscopy, peptide-conjugated lipid

## Abstract

Hemagglutinin (HA) plays an important role in the first step of influenza virus (IFV) infection because it initiates the binding of the virus to the sialylgalactose linkages of the receptors on the host cells. We herein demonstrate that a HA-binding peptide immobilized on a solid support is available to bind to HA and IFV. We previously obtained a HA-binding pentapeptide (Ala-Arg-Leu-Pro-Arg), which was identified by phage-display selection against HAs from random peptide libraries. This peptide binds to the receptor-binding site of HA by mimicking sialic acid. A peptide-conjugated lipid (pep-PE) was chemically synthesized from the peptide and a saturated phospholipid. A lipid bilayer composed of pep-PE and an unsaturated phospholipid (DOPC) was immobilized on a mica plate; and the interaction between HA and the pep-PE/DOPC membrane was investigated using atomic force microscopy. The binding of IFV to the pep-PE/DOPC membrane was detected by an enzyme-linked immunosorbent assay and real-time reverse transcription PCR. Our results indicate that peptide-conjugated lipids are a useful molecular device for the detection of HA and IFV.

## Introduction

Type A influenza virus (IFV) is highly mutagenic, and, thus, often causes epidemics and pandemics. IFV has been classified into subtypes based on the antigenic specificity of the envelope glycoproteins hemagglutinin (HA) and neuraminidase (NA; Gamblin and Skehel, 2010). Although, sixteen HA subtypes (H1-H16) and nine NA subtypes (N1-N9) have been identified to date, only three HA subtypes (H1, H2, and H3) and two NA subtypes (N1 and N2) have so far been identified among human pandemics. In order to prevent the spread of influenza, it needs to be diagnosed influenza quickly and easily in clinical practice by the detection of IFV.

In recent decades, several methods have been developed to detect IFV. Immunochromatography, PCR analyses, enzyme-linked immunoassays, and fluorescence antibody methods are frequency employed for clinical use (Eisfeld et al., 2014) and the surveillance of influenza (Wang and Taubenberger, 2010). On the other hand, a glycan array using oligosaccharides is used for basic research on the carbohydrate recognition of proteins (Feizi et al., 2003; Feizi, 2013). Although all methods have important characteristics, they also have some limitations. Antibodies, which are excellent biological components, are unstable and their production cost is high, PCR methods require expensive equipment and reagents, and the preparation of various kinds of oligosaccharides requires a large amount of work. In order to prevent global pandemics, easy, quick, and inexpensive methods are required to detect influenza.

The first step in the infection of cells by IFV is the binding of HA to sialic acid-containing glycoconjugates (Skehel and Wiley, 2000). Human HA strains preferentially bind to the terminal sialylgalactose structure (Neu5Acα2−6Gal), whereas avian and equine strains bind to the corresponding Neu5Acα2−3Gal (Suzuki, 2005). We previously identified the HA-binding pentapeptide, Ala-Arg-Leu-Pro-Arg, from phage-displayed peptide libraries (Matsubara et al., 2010). This pentapeptide binds to the receptor-binding site of HA by mimicking sialic acid, and exerts the inhibitory effects on IFV infection in cells. Furthermore, dendrimers composed of the peptide exhibit strong high inhibitory activities against IFV infection (Hatano et al., 2014). This peptide has potential in the capture of IFV with high sensitivity and specificity.

In the present study, we developed an artificial raft-like membrane to capture IFV using the HA-binding pentapeptide. IFV receptors such as ganglioside GM3 exist in membrane rafts, lipid domains that are 10–200 nm in diameter and act as a role of platform for protein-lipid interactions (Suzuki et al., 1986; Pike, 2006). In order to immobilize a solid surface, we designed and synthesized a peptide-conjugated lipid (pep-PE) composed of the peptide and dipalmitoylphosphatidylethanolamine (DPPE) using click chemistry (Tornøe et al., 2002). The pep-PE-containing lipid bilayer was immobilized on a mica plate, and influenza HA interacted with the pep-PE membrane. The surface topography of the pep-PE immobilized membrane was investigated using atomic force microscopy (AFM) and the results obtained indicated that HA selectively bound to the membrane. Furthermore, the binding of IFV was detected by ELISA, and the number of virus on the membrane was estimated by real-time reverse transcription PCR (rRT-PCR). These results suggest that the peptide-conjugated lipid designed in this study is useful as a novel device for the detection of IFV.

## Materials and methods

### Materials

DPPE was purchased from Wako Pure Chemical Industries, Ltd. (Japan). Dioleoylphosphatidylcholine (DOPC) and 1-palmitoyl-2-oleoyl-*sn*-glycelo-3-phosphocholine (POPC) were purchased from Sigma-Aldrich Co., LLC (St. Louis, MO, USA). Ganglioside Neu5Acα2–3Galβ1–4Glcβ1–1′Cer (GM3) from the bovine brain was purchased from Hytest Ltd. (Finland). Influenza hemagglutinins (HAs) derived from A/New Caledonia/20/99 (H1N1) and A/New York/55/2004 (H3N2) viruses were provided by Yujiro Suzuki (The Kitasato Institute, Japan; Matsubara et al., 2010). The human IFV strain A/Puerto Rico/8/34 (H1N1) was provided by Dr. Kyosuke Nagata (University of Tsukuba, Japan; Matsubara et al., 2009).

### Synthesis of an azide-containing pentapeptide (1)

A sialic acid-mimic pentapeptide with the ability to bind to the receptor-binding site of HA was previously identified (Matsubara et al., 2010). An azide group-conjugated peptide amide, Ala-Arg-Leu-Pro-Arg-Lys(N_3_)-NH_2_, was prepared by solid-phase peptide synthesis using standard 9-fluorenylmethoxycarbonyl (Fmoc) chemistry (Matsubara et al., 2009). Briefly, Fmoc-Lys(N_3_)-OH was loaded onto Fmoc-NH-SAL Resin (Watanabe Chemical Industries, Ltd., Japan), and the peptide was elongated manually in multiple batches on a 0.1-mmol scale. In order to cleave the peptide from the resin, the resin (0.1 mmol) was treated with 1 mL of a cleavage cocktail (trifluoroacetic acid/water/triisopropylsilane, 95:2.5:2.5 by volume) on ice for 2 h (Schneggenburger et al., 2010). The crude peptide was purified by high-performance liquid chromatography (HPLC), and the fraction of the product was lyophilized. Purity (>98%) and the expected structure were verified by HPLC and matrix-assisted laser desorption ionization/time-of-flight mass spectrometry (MALDI-TOF MS). Ala-Arg-Leu-Pro-Arg-Lys(N_3_)-NH_2_ **1** (16 mg, 21%): MALDI-TOF MS (*m/z*: calcd exact mass for C_32_H_60_N_16_O_6_ [M+H]^+^ 765.5, found 765.8).

### Synthesis of pentynoyl-DPPE (2)

In order to obtain an alkyne-modified lipid, DPPE was conjugated with 4-pentynoic acid using 4-(4,6-dimethoxy-1,3,5-triazin-2-yl)-4-methylmorpholinium chloride (DMT-MM) as a coupling reagent (Watanabe et al., 2004). DPPE (0.01 mmol), 4-pentynoic acid (0.03 mmol), and DMT-MM (0.01 mmol) were stirred at 25°C for 12 h in a mixture of chloroform (3 mL)/methanol (0.2 mL)/triethylamine (0.05 mL). The product was extracted with chloroform/sodium hydrogen carbonate solution, and solvents of the organic layer were evaporated. *N*-(4-pentynoyl)-DPPE **2** (7.2 mg, 90%) was obtained as oil: *R*_*f*_ 0.5 [8:2 (v/v) CHCl_3_–MeOH]; MALDI-TOF MS (*m/z*: calcd exact mass for C_42_H_78_NO_9_P [M+Na]^+^ 794.5, found 794.4).

### Synthesis of peptide-conjugated DPPE (pep-PE)(3)

Azide-containing peptide **1** (0.2 μmol), pentynoyl-DPPE **2** (0.4 μmol), CuSO_4_ (1.6 μmol), sodium ascorbate (1.0 μmol), and tris(benzyltriazolylmethyl)amine (TBTA)(0.2 μmol) were stirred at 25°C for 3 h in 1 mL of 50% methanol (Tornøe et al., 2002). The reaction was stopped on ice and the crude product was purified by HPLC on an ODS column with a liner gradient of 20–60% acetonitrile in 0.1% TFA. After lyophilization, purity (>98%) and the expected structure were verified by HPLC and MALDI-TOF MS. Peptide-conjugated DPPE (pep-PE) **3** (0.19 mg, 63%): MALDI-TOF MS (*m/z*: calcd exact mass for C_74_H_138_N_17_O_15_P [M+K]^+^ 1575.0, found 1576.2).

### AFM measurements of lipid bilayers

In order to investigate the surface topography of the lipid bilayer, lipid bilayers were prepared on mica as described previously (Iijima et al., 2009; Matsubara et al., 2013). Briefly, a POPC lipid monolayer at an air–water interface was prepared on a Langmuir–Blodgett trough at 25°C with a subphase of water, and transferred to freshly cleaved mica (1 × 1 cm) by horizontal deposition at a surface pressure of 35 mN m^−1^ (POPC-coated mica). A second lipid monolayer of pep-PE (or DPPE, DOPC, GM3, or pep-PE/DOPC) was loaded onto POPC-coated mica by horizontal deposition at a surface pressure of 30 mN m^−1^ to give a lipid bilayer.

The lipid bilayer was incubated with HA solution for 1 h at 25°C to observe the binding of H1 HA. After washing three times with phosphate-buffered saline (PBS), the lipid monolayer was subjected to AFM measurements.

AFM measurements of lipid bilayers on mica were carried out using SPM-9600 (Shimadzu Co., Japan) in water at 25°C. A 38-μm-long soft cantilever (BL-AC40TS-C2, Olympus) with integrated pyramidal silicon nitride tips having a spring constant of 0.1 Nm^−1^ was used for all measurements.

A number of topographic images were taken in the dynamic mode at a scanning rate in the range of 1–2 Hz, and the occupied area of target domains was estimated from typical multiple images (*n* = 3, 1 × 1 μm). In the estimation of domains, AFM images were binarized on the basis of the heights of the membrane, and pixels were counted using image processing software (Adobe Photoshop Elements). For example, the white area of a binarized image by thresholding at 8 nm from the bottom was identified as the area of the HA-bound domain of the pep-PE membrane (**Figure 3**).

### Enzyme-linked immunosorbent assay (ELISA)

A lipid monolayer of pep-PE/DOPC (50:50) or DOPC was directly loaded onto a few dozen plastic plates (13.5 mm in diameter; code 174950, Nalge Nunc international) by horizontal deposition at a surface pressure of 30 mN m^−1^. IFV [800–5600 plaque-forming units (pfu)] in 0.2 mL of PBS was incubated at 25°C for 1 h in pep-PE/DOPC- or DOPC-transferred plastic plates. The plates were washed three times with PBS, and their contents were then transferred into a 24-well plates that were blocked with 5% bovine serum albumin (BSA)/PBS at 4°C overnight.

The IFV-bound plates were incubated with a 1:1000 (v/v) dilution of an anti-hemagglutinin (A/H1N1) antibody (RayBiotech Inc.) for 1 h, and the primary antibody was then labeled with a 1:1000 (v/v) dilution of a peroxidase-conjugated anti-mouse IgG antibody (Merck Millipore) for 1 h. Color was developed using *o*-phenylenediamine, and changes in absorbance (Δ*A*) at 492 nm were determined by a microplate reader. Each experiment was performed in triplicate.

### rRT-PCR

The pep-PE/DOPC (50:50) lipid monolayer was attached horizontally to plastic plates as described above. IFV (H1N1, A/Puerto Rico/8/34; 1600 pfu in 0.14 mL of PBS) was incubated at 25°C for 1 h on the pep-PE/DOPC-transferred plastic plates. After washing with PBS, plates were incubated with AVL viral lysis buffer (QIAamp Viral RNA Mini Kit, QIAGEN) for 10 min. Viral RNA was extracted according to the manufacturer's instructions.

One-step RT-PCR was performed using QuantiTect SYBR Green PCR Kit (QIAGEN) with forward and reverse primers for the sequences of the matrix protein (M) gene for 244 bp (forward M30F2/08: 5′-ATGAGYCTTYTAACCGAGGTCGAAACG-3′; reverse M264R3/08: 5′-TGGACAAANCGTCTACGCTGCAG-3′; Eisfeld et al., 2014). The reaction was performed using a PikoReal Real-Time PCR system (TCR0096, Thermo Scientific). PCR was set up in a 10-μL reaction volume containing 5 μL of 2 × QuantiTect SYBR Green RT-PCR master mix, 0.1 μL of QuantiTect RT Mix, 0.5 μL of RNA template, 1 μL of forward and reverse primers (10 μM each), and 3.4 μL of RNase free water. The optimized cycling conditions were as follows: RT reaction at 50°C for 2 min, 95°C for 15 min, initial denaturation at 95°C for 30 s, followed by 50 cycles of denaturation at 95°C for 5 s, primer annealing at 57°C for 20 s, and extension at 72°C for 10 s. Fluorescence was measured at the end of each cycle. A melt curve analysis was performed following amplification in order to verify the specificities of the amplified products. A melting curve analysis consisted of 60°C for 30 s, and followed by a temperature increase to 95°C for 10 s with the continuous reading of fluorescence. The amplified products were analyzed by agarose gel electrophoresis (2.5% agarose) and detected by staining with ethidium bromide (Supplementary Figure 3A).

A series of two-fold dilutions of the virus solution starting from 400 to 3200 pfu were prepared in order to construct a standard curve. A liner regression relationship was observed between the amount of the virus and threshold cycle (*C*_t_) values with a coefficient of determination (*R*^2^) of 0.954 (Supplementary Figure 3B). The amount of virus that remained on the pep-PE/DOPC (50:50) membrane was estimated from the standard curve.

## Results

### Synthesis of peptide-conjugated DPPE

**Figure 1:**
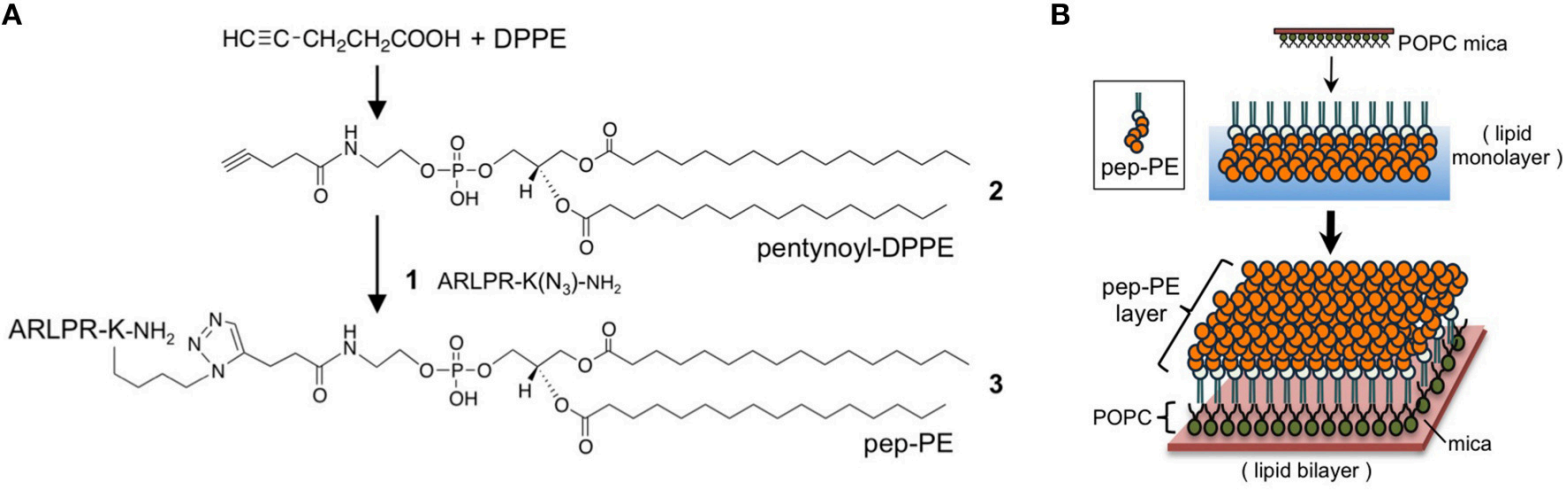
**Preparation of peptide-conjugated lipid membrane**. **(A)** Synthesis of a peptide-conjugated lipid. Alkyne-modified dipalmitoylphosphatidylethanolamine (DPPE) was prepared from 4-pentynoic acid and DPPE. The click reaction of the *N*-(4-pentynoyl)-DPPE (pentynoyl-DPPE, (**2**) and an azide-containing pentapeptide (**1**) gave peptide-conjugated DPPE (pep-PE, **3**). ARLPR, Ala-Arg-Leu-Pro-Arg. **(B)** Procedure of construction of pep-PE lipid membrane. A pep-PE monolayer was transferred to the POPC-coated mica to give the pep-PE lipid bilayer.

The sialic acid-mimic pentapeptide (Ala-Arg-Leu-Pro-Arg; Matsubara et al., 2010) was conjugated with DPPE by click chemistry to immobilize the HA-binding peptide on the lipid membrane (Figure 1A). Prior to the click reaction, the pentapeptide was modified with an azide group through the side chain of Lys (Ala-Arg-Leu-Pro-Arg-Lys(N_3_)-NH_2_, **1**; Supplementary Figure 1A). On the other hand, DPPE was modified with 4-pentynoic acid to give *N*-(4-pentynoyl)-DPPE (**2**, Supplementary Figure 1B). The click reaction of **1** and **2** quantitatively gave peptide-conjugated DPPE (pep-PE, **3**), and pep-PE was purified by HPLC and determined by MALDI-TOF MS (Supplementary Figure 1C).

### Binding of H1 HA to the pep-PE membrane

**Figure 2:**
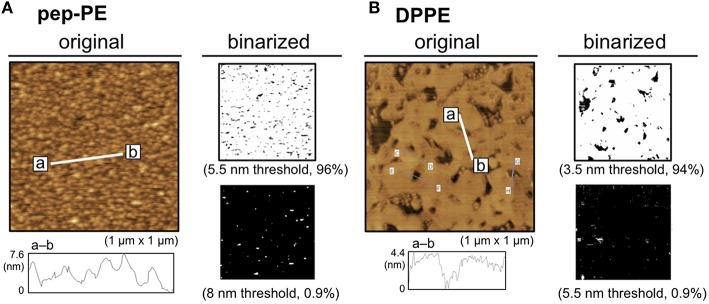
**Surface topographic studies of pep-PE and DPPE membranes by AFM**. **(A)** AFM image (original) and binarized AFM images (binarized) of the pep-PE membrane. In order to illuminate the area of higher lipid domains, an original AFM image (*left*) was binarized on the basis of the heights of the membrane (5.5 and 8 nm thresholds; *right*). **(B)** AFM image (original) and binarized AFM images (binarized) of the DPPE membrane. The original AFM image (*left*) was binarized on the basis of the heights of the membrane (3.5 and 5.5 nm thresholds; *right*).

A lipid monolayer of pep-PE was loaded onto POPC-coated mica to give a lipid bilayer that exposed the pep-PE layer (pep-PE membrane, Figure 1B; Matsubara et al., 2013). The surface topography of the pep-PE membrane was observed by AFM, and revealed that the height of the lipid domain was ~5.5 nm (Figure 2A). On the other hand, the height of the DPPE domain was ~3.5 nm (Figure 2B); therefore, the height of pep-PE was higher than that of DPPE. The difference in height (2.0 nm) was attributed to the size of the pentapeptide.

**Figure 3:**
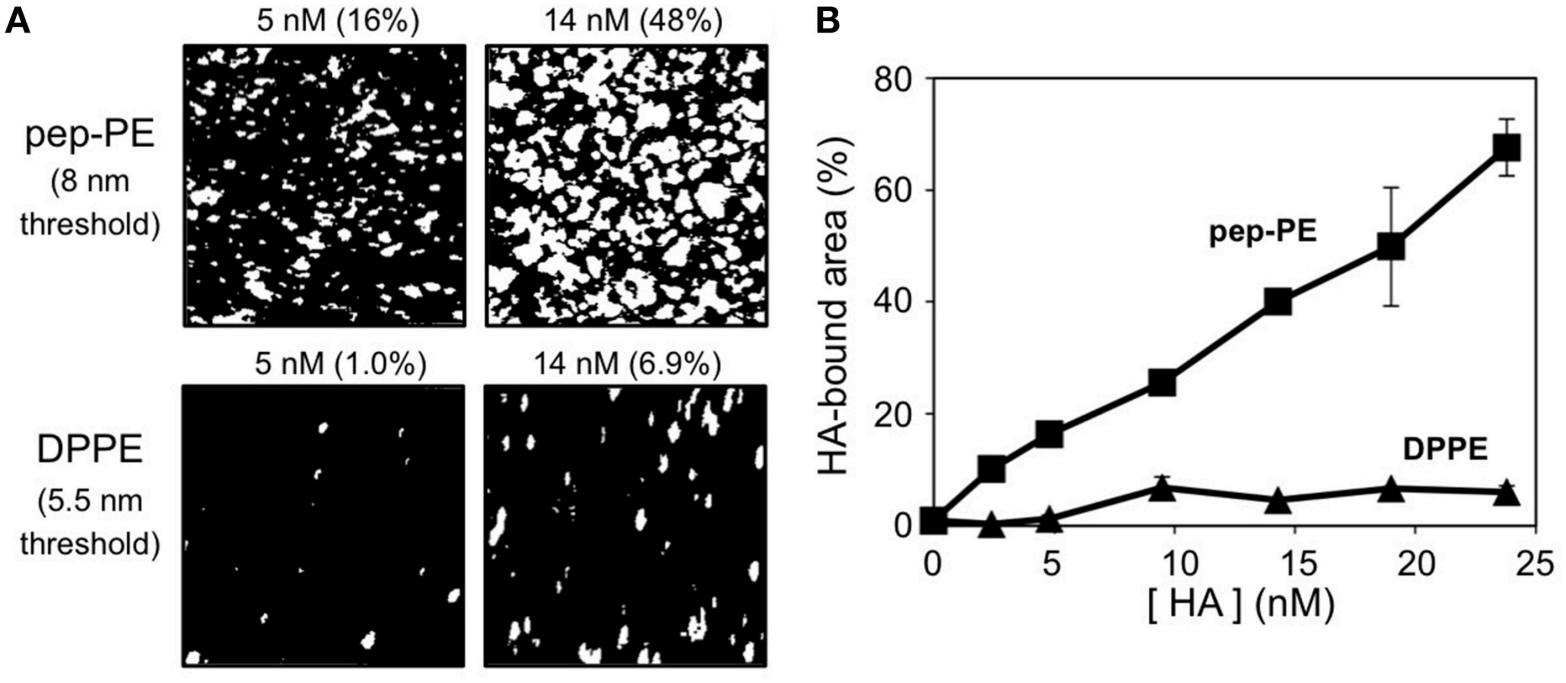
**Concentration-dependent binding of H1 HA on the pep-PE membrane**. **(A)** Binarized images of HA-bound pep-PE and DPPE membranes. AFM images were taken after the H1 HA incubation at 25°C for 1 h ([HA] = 2.4–24 nM). In order to illuminate the HA-bound area, original AFM images were binarized on the basis of the heights of the membranes (8 or 5.5 nm threshold). The percentage of white pixels in binarized images was estimated as the HA-bound area. **(B)** The HA-bound area was plotted as a function of HA concentration (H1 HA, *n* = 3).

HA of the H1N1 strain (A/New Caledonia/20/99) was interacted with the pep-PE membrane at 25°C for 1 h to investigate the binding of influenza HA, and the surface topography was observed by AFM. In order to illuminate the HA-bound area, the original AFM image was binarized on the basis of the height of the membrane (8 nm threshold) because the height of the bare pep-PE membrane was not greater than 8 nm (Figures 2A, 3A). The percentage of white pixels in binarized images was regarded as the HA-bound area. The HA-bound area increased in proportion to HA concentrations, and 65% of the membrane surface was covered at 24 nM of HA (Figure 3B). On the other hand, HA showed no significant binding to the DPPE membrane (10% or lower). These results indicated that the binding of HA was responsible for the sialic acid-mimic peptide of pep-PE.

### Binding of H1 HA to the GM3 membrane

**Figure 4:**
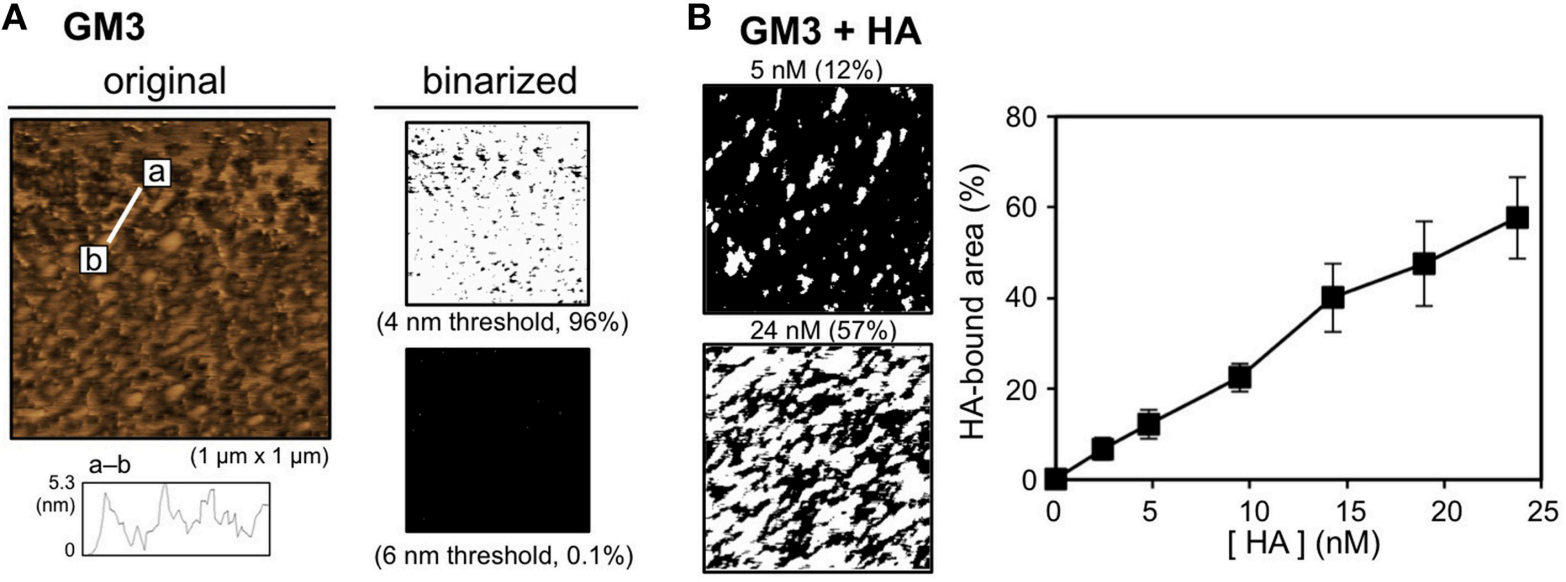
**Surface topographic study and the binding of H1 HA on the GM3 membrane**. **(A)** AFM image (original) and binarized AFM images (binarized) of the GM3 membrane. The original AFM image (*left*) was binarized on the basis of the heights of the membranes (4 and 6 nm thresholds; *right*). **(B)** Concentration-dependent binding of H1 HA on the GM3 membrane. AFM images were taken after the H1 HA incubation at 25°C for 1 h ([HA] = 2.4–24 nM). Binarized images of the HA-bound GM3 membrane at [HA] = 5 and 24 nM (6 nm threshold; *left*). The HA-bound area was plotted as a function of HA concentration (H1 HA, *n* = 3; *right*).

The ganglioside GM3 is an IFV receptor because HA binds to GM3 through the sialyllactose structure (Suzuki et al., 1986; Sato et al., 1996). The binding of HA to the GM3 membrane was investigated using AFM as well as pep-PE. The lipid bilayer that exposed the GM3 layer (the GM3 membrane) was prepared, and its surface topography was then observed by AFM. GM3 was similar in height to DPPE, at ~4 nm (Figure 4A). After the H1 HA incubation, the HA-bound area was estimated by the binarization of AFM images (6 nm threshold; Figure 4B, *left*). Similar to pep-PE, the HA-bound area increased in proportion to HA concentrations (Figure 4B, *right*). These results indicated that pep-PE has potential as an alternative to capture HA instead of GM3.

### Construction of the pep-PE/DOPC membrane

Glycosphingolipids (GSLs) such as gangliosides and neutral GSLs are enriched in the membrane microdomain, e.g., membrane (lipid) raft, of animal cell membranes (Pike, 2006). The membrane microdomain is one of the functional units in the membrane, and is considered to contribute to the many biological activities of lipids and proteins (Simons and Ikonen, 1997). The phase separation of the (glyco) sphingolipid/cholesterol/PC ternary system occurs in the presence of phosphatidylcholine (PC) with unsaturated acyl chain(s) such as POPC (Simons and Ikonen, 1997; de Almeida et al., 2003). Phase separation is detectable by height differences between the three phases; solid-ordered (*s*_o_), liquid-ordered (*L*_o_), and liquid-disordered (*L*_d_) domains, by AFM imaging (Johnston, 2007). We previously investigated the interaction between wheat germ agglutinin (WGA) and a GM3-containing membrane composed of lipids extracted from mouse B16 melanoma cells (Iijima et al., 2009). Since the results of AFM indicated that WGA binds to the highest domain [*L*_o_ and/or *s*_0_ phase(s)], this domain was identified as the GM3-enriched area.

**Figure 5:**
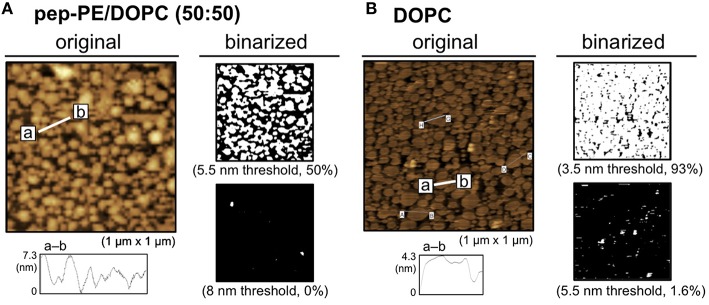
**Surface topographic studies of pep-PE/DOPC (50:50) and DOPC membranes by AFM**. **(A)** AFM image (original) and binarized AFM images (binarized) of the pep-PE/DOPC (50:50, molar ratio) membrane. Binarized images were generated by 5.5 and 8 nm thresholds. **(B)** AFM image (original) and binarized AFM images (binarized) of the DOPC membrane. Binarized images were generated by 3.5 and 5.5 nm thresholds.

Phase separation is responsible for differences in the phase transition temperatures (*T*_m_) of lipids; therefore, we have the ability to design model membranes composed of a low *T*_m_ lipid (e.g., unsaturated PC), high *T*_m_ lipid (a saturated PC or a sphingolipid), and cholesterol (de Almeida et al., 2003). In the present study, DOPC was mixed with pep-PE to prepare a membrane raft-like domain, and the binding of HA was investigated. A lipid monolayer of a mixture of pep-PE and DOPC (50:50, molar ratio) was loaded onto POPC-coated mica to give a lipid bilayer that exposed the pep-PE/DOPC layer (pep-PE/DOPC membrane). AFM images of the pep-PE/DOPC (50:50) membrane clearly showed phase separation, and the highest domain was estimated to be an area of 50% from a binarized image (5.5 nm threshold; Figure 5A). Since the height of DOPC was ~3.5 nm (Figure 5B), the highest domain in the pep-PE/DOPC (50:50) membrane was considered to be the pep-PE-containing domain.

### Binding of H1 HA to the pep-PE/DOPC membrane

**Figure 6:**
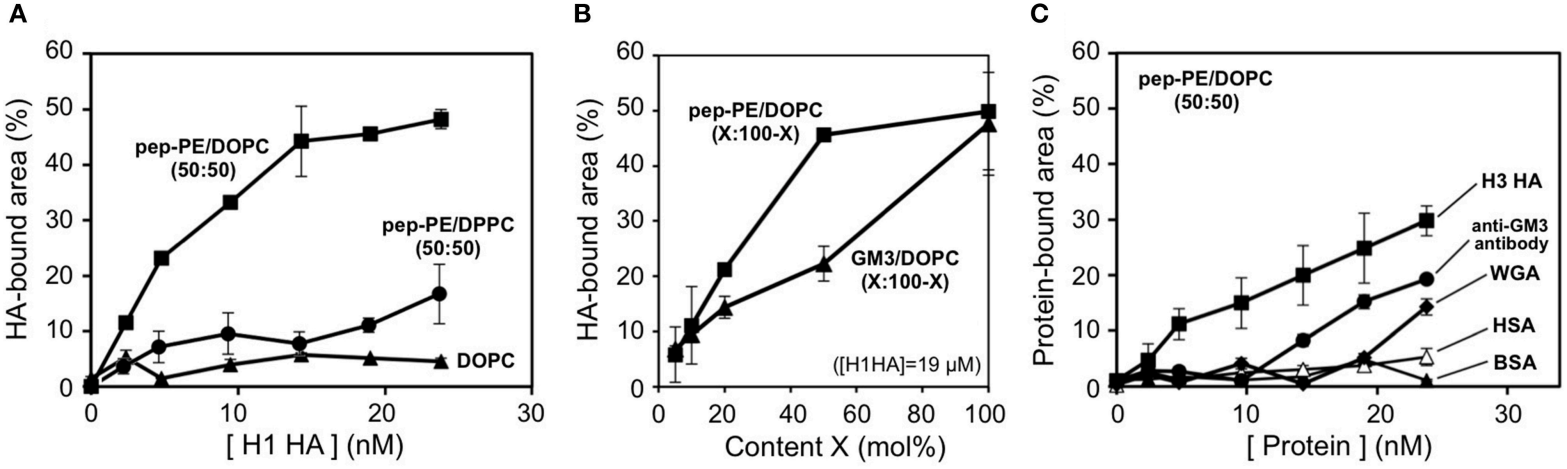
**Binding of HAs and proteins to the pep-PE/DOPC membrane**. **(A)** HA-bound areas of the pep-PE/DOPC (50:50), pep-PE/DPPC (50:50), and DOPC membranes were plotted as a function of HA concentration (H1 HA, *n* = 3). The HA-bound area was estimated from binarized AFM images (8 or 5.5 nm threshold, respectively). **(B)** HA-bound areas of the pep-PE/DOPC (X:100-X) and GM3/DOPC (X:100-X) membranes were plotted as a function of pep-PE and GM3 content X ([H1 HA] = 19 μM, *n* = 3). **(C)** Protein-bound areas of the pep-PE/DOPC (50:50) membrane were plotted as a function of protein concentration (*n* = 3). H3 HA, an anti-GM3 antibody, wheat germ agglutinin (WGA), human serum albumin (HSA), and bovine serum albumin (BSA) were incubated with the pep-PE/DOPC (50:50) membrane for at 25°C 1 h. The protein-bound area was estimated from binarized AFM images (8 nm threshold).

The HAs of H1 and H3 were interacted with the pep-PE/DOPC (50:50) membrane at 25°C for 1 h, and the HA-bound area was estimated from binarized images as described above (8 nm threshold). In the case of H1 HA, the HA-bound area showed a saturation curve against HA concentrations (Figure 6A). Although the content of pep-PE was reduced to 50 mol%, the HA-bound area of pep-PE/DOPC (50:50) membrane at around 5–20 nM was comparable to that of pep-PE membrane (Figure 3). The HA-bound area (49%) at 24 nM was similar to that of pep-PE domain (50%; Figure 5A), indicating that the pep-PE domain was covered with HA. Furthermore, in the case of the GM3/DOPC (50:50) membrane, the HA-bound area was significantly smaller (22%) than that of the GM3 membrane (48%; Figures 6B, 4B), suggesting the superiority of pep-PE over GM3 for HA binding.

On the other hand, the binding of HA to pep-PE/DPPC (50:50) was significantly less than that to pep-PE/DOPC (50:50; Figure 6A). A comparison with the AFM image of pep-PE/DOPC (50:50), as shown in Figure 5A, revealed the absence of the distinct phase separation of pep-PE/DPPC (50:50; Supplementary Figure 2). This was attributed to the *T*_m_ value of pep-PE being markedly different from that of DOPC (−3°C; Koster et al., 1994) and closer to that of DPPE (64°C; Ramezani et al., 2009) and DPPC (41.5°C; Parasassi et al., 1991). Hashizume et al. previously reported that an extensive affinity for lectin was induced by the phase separation of lactosylceramide (LacCer) in a LacCer/DOPC membrane (Hashizume et al., 1998). These findings suggest that the affinity of HA for a pep-PE-containing membrane increases with the formation of a raft-like domain in the presence of DOPC.

In order to investigate the interactions of pep-PE with other proteins, H3 HA, the anti-GM3 antibody, WGA, and two types of serum albumin were incubated with pep-PE/DOPC (50:50; Figure 6C). The amount of H3 HA that bound to the pep-PE/DOPC (50:50) membrane was lower than that of H1 HA (Figure 6A). An anti-GM3 antibody and WGA showed a lower amount of binding to the pep-PE/DOPC (50:50) membrane than HAs, and no binding was observed by human serum albumin (HSA) or bovine serum albumin (BSA) was observed (Figure 6C).

These results indicate that HA specifically interacts with the pep-PE/DOPC (50:50) membrane, and the composition of pep-PE/DOPC (50:50) has the potential to detect HA and IFV effectively.

### Capture of IFV by the pep-PE/DOPC membrane

**Figure 7:**
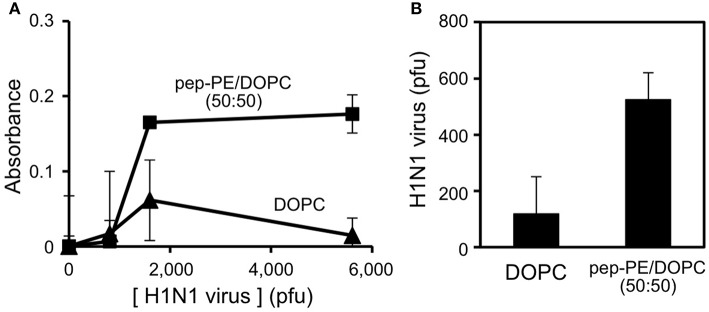
**Detection of IFV-binding to the pep-PE/DOPC (50:50) membrane by ELISA (A) and rRT-PCR (B)**. IFV (H1N1 strain) was interacted with the pep-PE/DOPC (50:50) membrane or DOPC membrane at 25°C for 1 h.

The binding of IFV of the H1N1 strain (A/Puerto Rico/8/34) to the pep-PE/DOPC membrane and DOPC membrane (as control) was evaluated by ELISA. IFV solutions containing 800, 1600, and 5600 pfu were incubated with membranes at 25°C for 1 h, and IFV that bound to the membranes was then detected. The absorbance of the pep-PE/DOPC (50:50) membrane was significantly increased at more than 1600 pfu (Figure 7A).

The amount of IFV that bound to the pep-PE/DOPC (50:50) membrane was quantified by rRT-PCR as described elsewhere (Tsukamoto et al., 2009). The specific amplification of the products (244 bp) from matrix protein (M) gene (Eisfeld et al., 2014) was confirmed by a melting curve analysis and agarose gel electrophoresis (Supplementary Figure 3A). A standard curve was constructed, and a linear regression relationship was observed between the amount of the virus (pfu) and the threshold cycle (*C*_t_) values was observed with a coefficient of determination (*R*^2^) of 0.954 and slope of −0.00199 (Supplementary Figure 3B). After the incubation of virus (1600 pfu) with the membrane for 1 h, 570 pfu of the virus was captured on the pep-PE/DOPC (50:50) membrane, and 100 pfu on the DOPC membrane (Figure 7B). These results indicate that IFV selectively binds to pep-PE-containing membranes.

## Discussion

Sialyloligosaccharide-containing compounds such as ganglioside GM3 are selected in order to capture HA and IFV on a solid support and are applied for immobilization onto microplates, (Totani et al., 2003) thin-layer chromatography plates, (Suzuki et al., 2000) the gold electrode of quartz-crystal microbalances, (Sato et al., 1996) the sensor chip of surface plasmon resonances, (Hidari et al., 2007) glycan microarrays, (Smith and Cummings, 2014) and nanoparticles, (Jannetto et al., 2010). Various (sialyl) glycoconjugates are needed to conduct these methods. (Koeller and Wong, 2001; Feizi et al., 2003) We have proposed the application of peptides with the ability to bind to lectin instead of glycoconjugates (Matsubara et al., 2010; Matsubara, 2012; Hatano et al., 2014). Such peptides bind to the receptor-binding site by mimicking (sialyl) oligosaccharide structures.

In the present study, we designed a peptide-conjugated lipid with the ability to play the role of gangliosides in the cell membrane. A sialic acid-mimic pentapeptide was conjugated to DPPE by click chemistry (Figure 1; Tornøe et al., 2002) and peptide-conjugated DPPE (pep-PE) was used as a constituent of the lipid bilayer (Figure 2). Pep-PE-containing lipid bilayers were prepared and immobilized on mica or plastic plates for AFM (Figures 3, 5). When pep-PE co-existed with an unsaturated PC, the pep-PE-containing membrane (pep-PE/DOPC, 50:50) exhibited useful affinity for HA and IFV (Figures 6, 7). AFM images indicated that pep-PE/DOPC membrane forms pep-PE-enriched domains (Figure 5), and this excellent affinity for HA and IFV is due to domain formation. This domain is known as a membrane microdomain (membrane raft), and we successfully constructed an artificial lipid bilayer composed of peptide-conjugated lipids without GSLs. Furthermore, ELISA and rRT-PCR indicated that IFV selectively bound to the pep-PE-containing membrane (Figure 7).

A comparison with ganglioside GM3 revealed that the ability of pep-PE to capture HA and IFV. The amount of HA that bound to the pep-PE membrane was similar to that of HA to the GM3 membrane (Figures 3, 4). On the other hand, the affinity of HA for the pep-PE-containing membrane was improved by the co-existence of DOPC (pep-PE/DOPC, 50:50; Figure 6). These results indicate that pep-PE is superior to GM3 for the binding of HA and IFV to mixed lipid membranes.

Sialylglycoconjugates are valuable compounds, and specific techniques and extensive efforts are required for their organic synthesis and isolation from natural sources (Koeller and Wong, 2001; Feizi et al., 2003). In order to detect proteins and pathogenic materials, glycoconjugates are immobilized on solid supports after biotinylation or another derivatization (Angus et al., 2000; Grün et al., 2006). There are several advantages to using the peptides described in the present study instead of glycoconjugates such as ganglioside GM3: (1) Functional peptides may be obtained by a *de novo* design and affinity selection system including phage-display technology, (Ladner et al., 2004) (2) since peptides are chemically stable, they may be stored for long periods of time, and (3) procedures to produce peptides have already been established (e.g., chemical synthesis) such that peptides are produced cheaply and in large quantities (Bray, 2003). As shown in the present study, the peptide-conjugated lipid was easy to synthesize using a click reaction. Instead of glycoconjugates, artificial peptides that mimic glycoconjugates are considered to be applicable to the capture of glycan-related proteins and pathogenic materials.

## Conclusion

In the present study, we showed that HA and IFV are detectable using a HA-binding peptide-conjugated lipid. This peptidyl lipid was able to be prepared for a lipid bilayer, and the affinity of HA was improved by domain formation in the presence of unsaturated PC. If a sufficient variety of sugar-mimic peptides is designed, these peptide-conjugated lipids may be used in addition to GSLs. The synthesis of the peptide-conjugated lipid is easier than that of glycolipids. Along with other glycoconjugates, sugar-mimic peptides and peptide-conjugated lipids have the ability to immobilize on nano- and microplates, nanoparticles, and other materials. In addition to IFV, peptide-conjugated lipids have the potential to detect biomolecules, toxins, viruses, and pathogenic materials. Peptide-conjugated lipids may be useful not only for the diagnosis and surveillance of influenza, but also those of other sugar-related diseases.

## Author contributions

TS conceived the project and TM and TS designed the research. RS and TM conducted experiments, and all authors discussed the results and implications. TM and TS prepared the manuscript.

### Conflict of interest statement

The authors declare that the research was conducted in the absence of any commercial or financial relationships that could be construed as a potential conflict of interest.
