# Supplementary material for: Binding of Hemagglutinin and Influenza Virus to a Peptide-Conjugated Lipid Membrane
Source: Front Microbiol. 2016 Apr 7;7:468. doi: 10.3389/fmicb.2016.00468 (PMC4823272; doi:10.3389/fmicb.2016.00468)
Supplement: Supplementary file 1 [file DataSheet1.DOCX]

Supplementary Material

Binding of hemagglutinin and influenza virus to a peptide-conjugated lipid membrane

**Teruhiko Matsubara, Rabi Shibata, Toshinori Sato***

*** Correspondence:** Toshinori Sato: sato@bio.keio.ac.jp


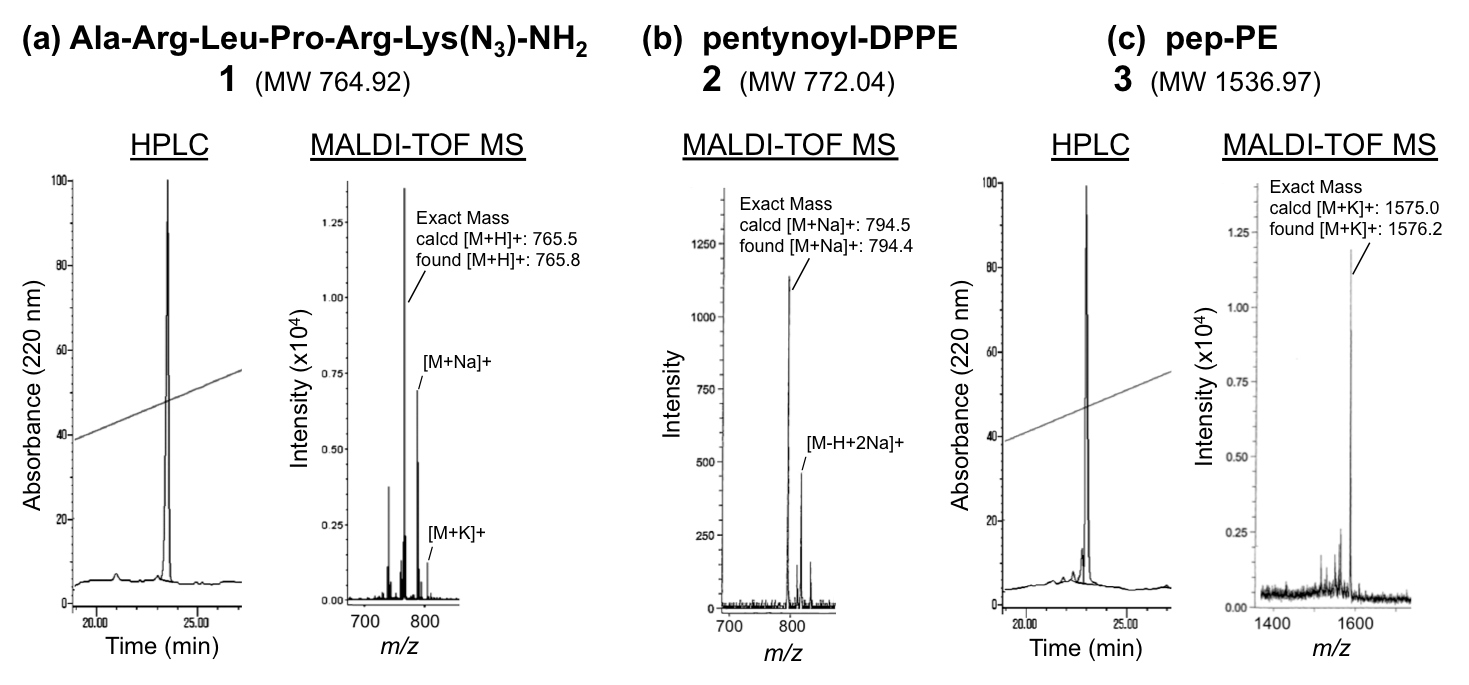


**Supplementary Figure 1.** **HPLC chromatograms and MALDI-TOF MS charts of Ala-Arg-Leu-Pro-Arg-Lys(N_3_)-NH_2_ (1)(a), *N*-(4-pentynoyl)-DPPE (2)(b), and peptide-conjugated DPPE (pep-PE, 3)(c).**


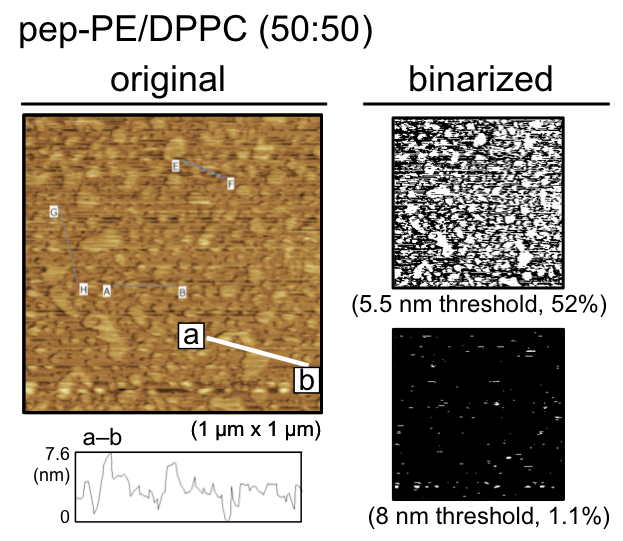


**Supplementary Figure 2.** **Surface topographic studies of pep-PE/DPPC (50:50) membrane by AFM.** AFM image (original) and binarized AFM images (binarized) of the pep-PE/DPPC (50:50, molar ratio) membrane. Binarized images were generated by 5.5 nm and 8 nm thresholds.


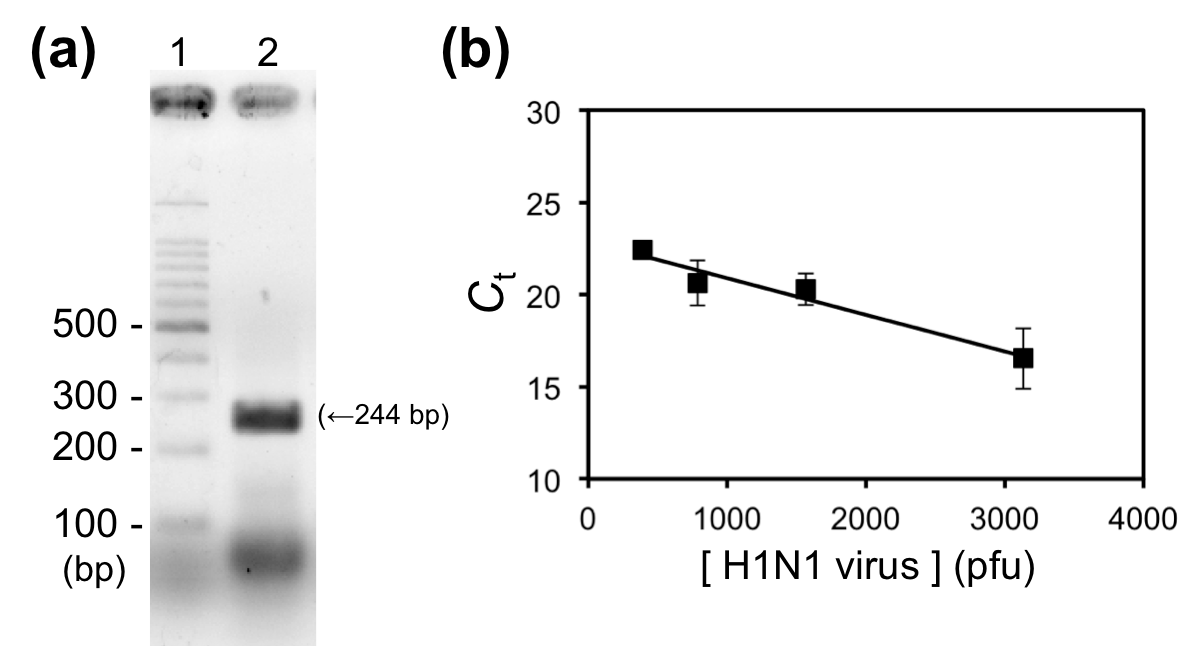


**Supplementary Figure 3.**  **Estimation of the number of IFV bound to the pep-PE membrane.** (a) Agarose gel electrophoresis of the PCR product from matrix protein (M) gene. lane 1, 100 bp DNA ladder; lane 2, PCR product. (b) Standard curve for IFV detection determined by rRT-PCR.
